# Supplementary material for: Inter-limb asymmetries are associated with decrements in physical performance in youth elite team sports athletes
Source: PLoS One. 2020 Mar 3;15(3):e0229440. doi: 10.1371/journal.pone.0229440 (PMC7053777; doi:10.1371/journal.pone.0229440)
Supplement: Attachment — Submitted filename: Response to reviewers.docx [file pone.0229440.s004.docx]

PONE-D-19-17934R1

Reviewers' comments:

**Reviewer #1: I would like to thank the authors for considering most of my previous comments. However, I still have some concerns. These are detailed below.**

**Title
Given that you did not directly measure any neuromuscular variables, the word "neuromuscular" seems to not reflect what was really done. Is there any other alternative that could be used here?**

Ok, we have changed the title and all document to: **“**Inter-limb asymmetries are associated with decrements in physical performance in youth elite team sports athletes”.

**Abstract**
**Line 35 Please add the magnitude of the correlation consistently in the abstract.**

Thanks, we clarified and added the magnitude of the correlation consistently. “Results showed significant (p ˂ 0.05) but small (r = 0.3) relationships between SLCMJ asymmetries and 30 m sprint time for the total group. Significant negative correlations with small to moderate magnitude of correlation were also found between SLCMJ asymmetries and SLCMJ performance on the lowest performing limb for the total group (p < 0.05; r = -0.3), males (p < 0.01; r = -0.5) and females (p < 0.05; r = -0.3). Moreover, significant negative correlations with moderate and large magnitude were also present between OLHT asymmetries and OLHT performance on the lowest performing limb for the total group (p < 0.01; r = -0.4), males (p < 0.01; r = -0.6) and females (p < 0.01; r = -0.6).”

**Line 36-39 Are these correlation analyses needed as one may assume that asymmetry is associated with the performance of the weaker leg. In other terms, why correlating asymmetry with a factor that was used to calculate asymmetry? It would make sense to examine the association between asymmetry and another measure of physical fitness but with the weaker leg, this is unclear to me.**

We agree with the author. Despite this, this sub-analysis reaffirms the relationship between the weakest leg and a decrease in the physical performance of athletes.

**Line 44 In the discussion you noted " a tendency". Please be consistent with the statements**
In the discussion we talked about “tendency” since the correlation is significant but the magnitude of this was low in some cases. We removed the adjective "tendency" to not confuse the reader.

**Introduction**
**Line 104 Please refer to the exact components of physical fitness used consistently.**

Added: “Therefore, the main objective of this study was to examine the relationship between inter-limb ~~neuromuscular~~ asymmetries and physical performance (Dynamic balance, jumping, linear sprinting, and change of direction speed) in elite youth team-sports players.”

**Line 110 See my previous comment.**

Added in the previous comment.

**Methods
Line 180 Please note that the jump height as a measure of power has been recently questioned in a comprehensive paper published in sports Med**

**doi: 10.1007/s40279-019-01073-1**

**You may mention this in the limitation**

We agree with the author. The ability to jump is not the only indicator of the power of the athlete. In this study we have used the jump because it is one of the main motor skills that determine performance in team sports such as volleyball, basketball or handball.

**Line 244 It is rather the magnitude of the difference**

Changed: “Moreover, the magnitude of the difference in asymmetry between males and females were determined using Cohen’s *d* effect sizes (ES) using the formula…”

**Results
Line 285-294 Please refer to my comment in the abstract**

We clarified the text: “Pearson’s correlations (*r*) between inter-limb asymmetry and test scores are shown in Table 3. Results showed significant (*p* ˂ 0.05) but small (*r* = 0.3) relationships between SLCMJ asymmetries and 30 m sprint time for the total group. Significant negative correlations with small to moderate magnitude of correlation were also present between SLCMJ asymmetries and SLCMJ performance on the lowest performing limb for the total group (*p* < 0.05; *r* = -0.3), males (*p* < 0.01; *r* = -0.5) and females (*p* < 0.05; *r* = -0.3). ~~However, the magnitudes of the correlations between SLCMJ asymmetries and SLCMJ performance on the lowest performing limb were small to moderate for the total group (~~*~~r~~* ~~= -0.3), females (~~*~~r~~* ~~= -0.3) and males (~~*~~r~~* ~~= -0.5).~~ Significant negative correlations with moderate and large margnitude were also present between OLHT asymmetries and OLHT performance on the lowest performing limb for the total group (*p* < 0.01; *r* = -0.4), males (*p* < 0.01;*r* = -0.6) and females (*p* < 0.01;  *r* = -0.6). In addition, no correlation were observed between SEBT ANT asymmetries and physical performance.”

**Discussion
Line 302-305 This particular statement is hard to grasp. Please rephrase for clarity**

We rephrased: **“**Results showed ~~a tendency~~ that larger jump height asymmetries during the SLCMJ test were associated with slower 30 m sprint times when the entire sample were considered. The same asymmetry index was also associated with reduced jump height on the lowest performing limb for the entire sample and when males and females were separated.”

**Line 305-307 Unclear**

We clarified: “Larger asymmetries during the OLHT were also associated with reduced distance during the OLHT~~, also~~ on the lowest performing limb for the whole sample and when separated by males and females.”

**310 However instead of despite**

Changed.

**313 consider starting a new sentence "This is in accordance....."**

Changed.

**319-321 awkward sentence. Please rephrase**

**Clarified: “**In contrast, in the present study, no significant associations were evident between jump height asymmetry and total time during the V-cut test.”

**321-325 What could be the reason behind this observation? why jump height asymmetry is associated with sprint speed performance but not with CoD performance. This is a comment that I included in my previous report but was not really considered.**

Clarified: “In contrast, in the present study, no significant associations were evident between jump height asymmetry and total time during the V-cut test. This is also in agreement with previous research. Lockie et al. (16) and Dos’Santos et al. (17) both showed no association between jump height and distance asymmetry from the SLCMJ and single and triple hop tests and COD (505) performance in male collegiate athletes. However, this is in contrast to previous research wich has shown realtionship between major inter-limb asymmetries and COD speed decrements [3,42]. Thus, the lack of consensus regarding the impact of inter-limb asymmetries on COD performance could be explained because of differences in the assessement methods, asymmetry calculations and athlete population.”

**329-332 This looks different from what you stated in the previous sentence.
you focused on the association between asymmetry and performance of the weaker leg (which I'm not that convinced why you did it or at least this has to be argued in the rationale) while Bishop et al reported on the correlation between asymmetry and jump performance without mentioning if it was relative to the weaker side or not.**

This is because Bishop et al. presented the data as left vs right (not stronger vs weaker).

**364 "In" instead of at**

Changed.

**383-384 Do you mean simply here that the level of asymmetry is more pronounced in females than that in males? if so would be better to express it that way.**

Clarified: “Despite this, our hypotheses was that the level of asymmetry would be more pronounced in females than that in males. However, our hypothesis has been disproven with no significant diffferences between groups in unilateral jumping tasks.”

**404 should be considered with caution instead**

Changed.

**Conclusions
424-425 Please reword**

“In addition, practitioners should also consider strengthening the weaker limb during sport-specific skills as well. For example, in a basketball game, performing a “lay-up” on the weaker side (weaker leg) successfully can benefit the performance of the player.”

**Reviewer #2: Dear authors, thank you for your modifications which improved the quality of your manuscript. Please add the following information to your text body.**

**- l.186: I do not understand why the OLHT should estimate "dynamic stability". Please
expand.**
Dynamic stability is defined as the ability to maintain equilibrium during dynamic
actions (the body is under some kind of displacement), bringing the requirement of
balance to the kind of joint stability involved in sport-specific skills. Here some
references (Noyes et al, 1996; Myer et al, 2011; Munro et al., 2012). We can add to the
text if the reviewer considers it necessary.

The stability component of the OLHT test is not in the jump capacity but in the stability that the lower limb should provide just after a maximum unilateral jump (subjects must hold the balance for two seconds after landing).

**- Table 3 and throughout the whole manuscript: I am not sure if the other reviewer really wanted that you use just one decimal for correlations. In my opinion, you should follow this rule of thumb: if you have 2 digits in front of the dot, use 1 decimal; if you have 1 digits in front of the dot, use 2 decimals. Please modify!**

We are totally agreed. But this is the comment of reviewer 1. “Would be better to round the values to one single decimal after the dot. Please, do this consistently throughout the manuscript.”. If the reviewer consider we have to change it is not a problem for us.
