# Supplementary material for: Inter-limb asymmetries are associated with decrements in physical performance in youth elite team sports athletes
Source: PLoS One. 2020 Mar 3;15(3):e0229440. doi: 10.1371/journal.pone.0229440 (PMC7053777; doi:10.1371/journal.pone.0229440)
Supplement: Attachment — Submitted filename: Response to reviewers.docx [file pone.0229440.s005.docx]

[PONE-D-19-17934R2] - [EMID:581e600bd4eca94c]

**Additional Editor Comments (if provided):**

**As for the decimals, please follow the recommendation of reviewer 2 (i.e. if you have 2 digits in front of the dot, use 1 decimal; if you have 1 digit in front of the dot, use 2 decimals)**

Changed.

Reviewers' comments:

**Reviewer #1**: **I thank the authors for responding to my comments. After reading the revision, I noted some further comments and suggestions.
Major concern
I have raised the concern about correlating the asymmetry with the performance of the weaker leg in my previous review report. I find authors’ answer not convincing in this context. I still think that the correlation analyses authors’ have made between asymmetry and the outcome of weaker side do not have a real significance. Above all, the weaker leg was used to determine asymmetry. Then, a significant correlation is not surprising.**

We agree with the reviewer and have added the following information to the limitations:

“Another finding of this study is that larger jump height asymmetries (vertical and horizontal directions) are associated with diminished jump performance; specifically in the weaker leg and appear to be direction-specific. However, these results do have some limitations that practitioners should be aware of. The significant correlation between asymmetry and the weaker leg is to be expected, given that asymmetry is ratio of both the stronger and weaker limbs. Therefore, it is suggested that correlations with ratios should be made with independent variables (e.g., jump height asymmetry vs. speed or CODS). Despite this, these results are in accordance with Bishop et al. (7) who also showed that larger jump height and distance asymmetries (from the SLCMJ and triple hop tests) were also associated with reduced jump performance during the SLCMJ and horizontal hop tests respectively [7]. In this case, they found decrements in performance in both limbs, which is contrary to our study, where significant correlations were noted in the weaker limb only. The differences in results between studies could be explained because Bishop et al. (7) presented the data as left vs. right (not stronger vs. weaker).

**Methods
Considering the criticisms related to the ability of jump height to predict muscle power, I would recommend to mention this point in the limitations of the study.**

Thanks for the consideration, but measure muscle power was not the aim of our study. We modified this sentence: “The one leg hop for distance is used to estimate unilateral jump capacity.”

**Discussion
Line 309: instead of tendency which was already removed, I suggest to note the range of magnitude (e.g., small-to-moderate)**

Added. “Results showed that larger jump height asymmetries during the SLCMJ test were associated (small-to-moderate magnitude) with lower 30 m sprint performance when the entire sample were considered.”

**Line 310: rather “lower 30 m sprint performance”. It may not be that clear to talk about “slower time”.**

Changed before.

**Line 333 spelling error
In a previous reply authors noted : “ this is because Bishop et al presented the data as left vs right (not stronger vs weaker)”. Sorry, may be I missed something, but I still do not get what you want to express. So, Bishop et al introduced the data as left vs right, then how can the reader knows the weaker from the stronger side?**

Please see our amendments above re: your additional major concern where hopefully this now has been addressed. Thank you for considering our stance on this.

**Reviewer #2**: **- l.186: I do not understand why the OLHT should estimate "dynamic stability". Please expand.
Dynamic stability is defined as the ability to maintain equilibrium during dynamic
actions (the body is under some kind of displacement), bringing the requirement of
balance to the kind of joint stability involved in sport-specific skills. Here some
references (Noyes et al, 1996; Myer et al, 2011; Munro et al., 2012). We can add to the
text if the reviewer considers it necessary.
The stability component of the OLHT test is not in the jump capacity but in the stability that the lower limb should provide just after a maximum unilateral jump (subjects must hold the balance for two seconds after landing).**
**--> As indicated in line 202 and Table 2, you analysed the greatest distance for OLHT. This is in my opinion no measure for dynamic stability. Please expand why you analysed the jump capacity, although you emphasize the stability component of OLHT. Please rephrase l.195! What happened if participants performed 2 invalid jumps with one leg?**

We agree with the author, we delete the component of dynamic stability in the text, this is not the aim of the test. “The one leg hop for distance is used to estimate unilateral jump capacity.” We also added. “If participants performed 2 invalid jumps with one leg, they had the opportunity to jump as many times as necessary until they kept their balance.”

**- l. 206: Please state where the front foot was placed in relation to the first timing gate.**

Added. “The front foot was placed 0.5 m before the first timing gate.”

- **Table 3 and throughout the whole manuscript: I am not sure if the other reviewer really wanted that you use just one decimal for correlations. In my opinion, you should follow this rule of thumb: if you have 2 digits in front of the dot, use 1 decimal; if you have 1 digits in front of the dot, use 2 decimals. Please modify!
We are totally agreed. But this is the comment of reviewer 1. “Would be better to round the values to one single decimal after the dot. Please, do this consistently throughout the manuscript.”. If the reviewer consider we have to change it is not a problem for us.
--> Please contact the editor to ask for journal guidelines concerning this issue.**

Thanks. Changed in all document.
